# Supplementary material for: Identification of photoperiod-induced specific miRNAs in the adrenal glands of Sunite sheep (Ovis aries)
Source: Front Vet Sci. 2022 Jul 22;9:888207. doi: 10.3389/fvets.2022.888207 (PMC9354845; doi:10.3389/fvets.2022.888207)
Supplement: Supplementary file 1 [file Data_Sheet_1.ZIP › Supplementary file/miRDeep2 file.zip!/miRDeep2 file/Novel_Structure/SPLP42A1/Novel_553.pdf]

Provisional ID	:	NC_019470.2_18793
Score total	:	2.4
Score for star read(s)	:	-1.3
Score for read counts	:	0
Score for mfe	:	2.1
Score for randfold	:	1.6
Score for cons. seed	:	
Total read count	:	20
Mature read count	:	20
Loop read count	:	0
Star read count	:	0

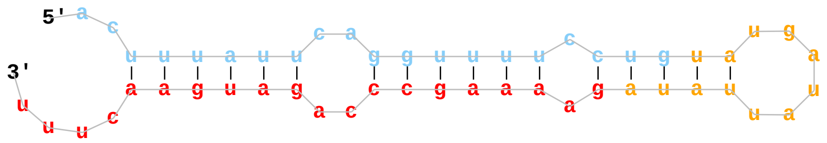

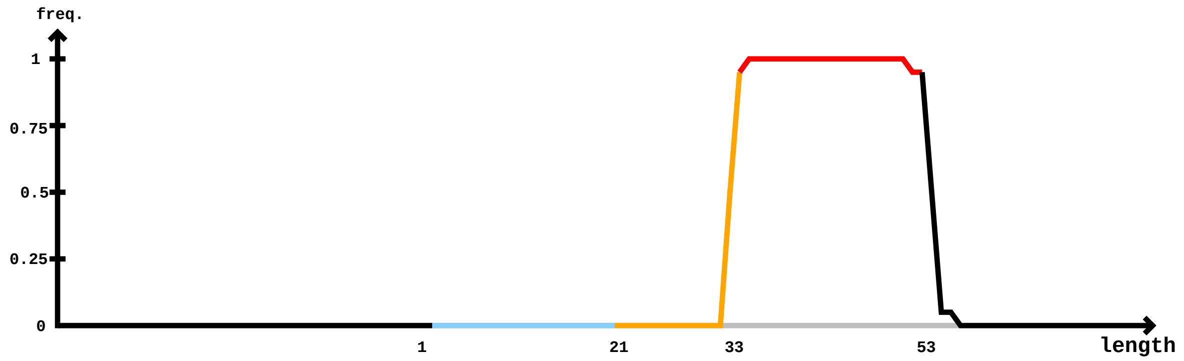

Star Mature

```
5'- cgccucuuggucauggcauauauuggguuggccaaaaacuuuauucagguuuuccuguaugauauuauagaaaagcccagaugaacuuuuuggccaacccaauaauuauuuu   -3'    exp
    .(((..........)))...((((((((((((((((((.((((((..((((((.(((((......))))).))))))..)))))).))))))))))))))))))........   reads      mm        sample
    ....................................................................agaaaaAcccagaugaacuuu.......................   3          1         seq
    ....................................................................agaaaaAcccagaugaacuuuu......................   7          1         seq
    .....................................................................gaaaaAcccagaugaacu.........................   1          1         seq
    .....................................................................gaaaaAcccagaugaacuuu.......................   8          1         seq
    ......................................................................aaaagcccagaugaacuuuuug....................   1          0         seq
```
